# Supplementary material for: Generation of a Transgenic Plasmodium cynomolgi Parasite Expressing Plasmodium vivax Circumsporozoite Protein for Testing P. vivax CSP-Based Malaria Vaccines in Non-Human Primates
Source: Vaccines (Basel). 2025 May 17;13(5):536. doi: 10.3390/vaccines13050536 (PMC12115629; doi:10.3390/vaccines13050536)
Supplement: Supplementary file 1 [file vaccines-13-00536-s001.zip › vaccines-3529602-supplementary.pdf]

A

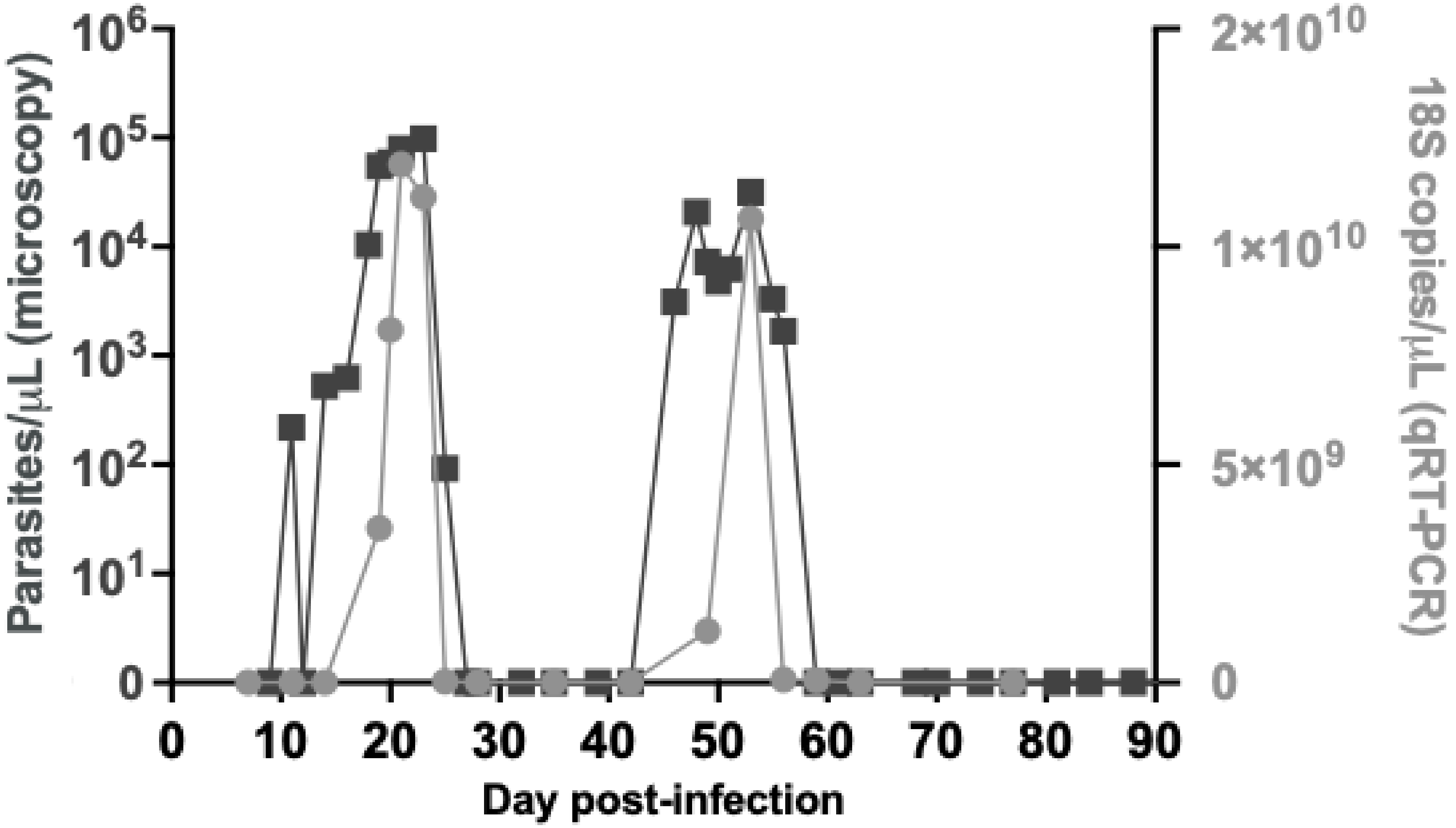

Supplemental Figure S1. RM3 parasitemia by qRT-PCR and microscopy across primary and relapse infections. Comparison of parasitemia as measured by microscopy (left axis) and qRT-PCR (right axis) quantified by comparison to a standard curve made of Armored RNA. qRT-PCR data points are the average of three triplicates.

Berok project primer table					
Primers for genotyping of transfectant parasites					
**Name**	**Species**	**Gene**	**F/R**	**Purpose**	**Sequence**
CJ388	PcBerok	CSP	F	Fwd upstream of 5' flank for genotyping Berok CSP chimera 789pb with CJ389	CATATCTGTACATGTCCATGTAGTGACC
CJ389	Pv	CSP	R	Rev for 5' integration in Berok	GCAGAATACTACTCACGGCGAGC
CJ390	PcBerok	CSP	R	Rev to detect wt in Berok 889 bp with CJ388	AGTGAACTGGCGTCTACATTATTGAAGC
CJ391	Pv	CSP	F	Fwd to detect Pv CSP in Berok	GTTATTCTGCTGGTGCTGGCCC
CJ392	Pc	CSP	F	Fwd to detect wt CSP in Berok	AAAACCAGAAGAGCTTGATGTGAATGACC
CJ393	Pc	CSP	R	Rev in Berok CSP region outside of 3' flank	ACGCAGTTTGCACACACCTGGC

Supplemental Table S1. Primers used for genotyping parasites
